# mGluR5 in amygdala modulates fear memory generalization

**DOI:** 10.3389/fnbeh.2023.1072642

**Published:** 2023-02-20

**Authors:** Shou-Min Xuan, Ya-Wen Su, Yi-Meng Liang, Zhen-Jie Gao, Chun-Yan Liu, Bu-Fang Fan, Yan-Wei Shi, Xiao-Guang Wang, Hu Zhao

**Affiliations:** ^1^Guangdong Province Translational Forensic Medicine Engineering Technology Research Center, Faculty of Forensic Medicine, Zhongshan School of Medicine, Sun Yat-sen University, Guangzhou, Guangdong, China; ^2^Guangdong Province Key Laboratory of Brain Function and Disease, Zhongshan School of Medicine, Sun Yat-sen University, Guangzhou, Guangdong, China

**Keywords:** fear conditioning, generalization, amygdala, GPCR, mGluR5

## Abstract

**Introduction:**

Fear memory generalization is regarded as the core characteristic of posttraumatic stress disorder (PTSD) development. However, the mechanism that contributes to the generalization of conditioned fear memory is still unclear. The generalization is generally considered to be a mismatch that occurs during memory consolidation.

**Methods:**

Foot shocks and tones were given as unconditioned stress and conditioned stress, respectively for fear conditioning training. Immunofluorescence staining, western blotting and qPCR were performed to determine the expression of different genes in amygdala of mice after fear conditioning training. Cycloheximide was used as a protein synthesis inhibitor and 2-methyl-6-phenylethynyl-pyridine was injected for mGluR5 inhibition.

**Results:**

Fear conditioning using caused incremental generalization, which was clearly observed during training. The density of c-Fos^+^ cells or the synaptic p-NMDAR expression did not differ with stress intensities. Strong-shock fear conditioning could induce significant mGluR5 de novo synthesis in the amygdala, which was not observed in the weak-shock group. Inhibition of mGluR5 impaired fear memory generalization induced by strong-shock fear conditioning, but the generalization level induced by weak-shock training was enhanced.

**Discussion:**

These results indicated that mGluR5 in the amygdala is critical to the function of inappropriate fear memory generalization and suggested that this may be a potential target for the treatment of PTSD.

## 1. Introduction

Post-traumatic stress disorder (PTSD) is a psychiatric disorder that occurs with delayed onset and persists over time after an individual suffered life-threatening stress. The core symptoms of PTSD are pathological re-experience, hyperarousal, and avoidance behaviors. Established theories suggest memory storage favor more gist-like representations rather than detailed events, a phenomenon referred to as memory generalization. Fear memory generalization enables animals to quickly and appropriately respond to novel stimuli that resemble a previous experience but make them show defensive reactions to a safe environment incorrectly in pathologic conditions (Fulton et al., 2015; Herringa, 2017; Compean and Hamner, 2019). Clinical psychopathologic studies have identified fear memory generalization as an important mechanism in the development of psychiatric disorders such as anxiety disorder, depressive disorder, and PTSD (Lissek et al., 2014). Previous studies on fear memory generalization were mainly based on the Pavlovian conditioning theory, using associative learning paradigms. In these paradigms, animals were trained to match neutral conditioned stimuli (CS) such as experimental environment (context) or given sounds (cues) with aversive unconditional stimuli (US) such as foot shock or blowing. After several times of associative learning, the neutral stimuli arouse similar negative emotional valence to that of the aversive stimuli (Phillips and LeDoux, 1992).

The amygdala, located in the middle of the temporal lobe, is thought to be the central hub for receiving somatosensory information. Especially in the associative learning paradigm, it was reported glutamatergic projections transmitted the combined signals of US and CS to neurons in the amygdala. The CS could selectively activate glutamatergic neurons, while the US provided a unified depolarizing signal that activated NMDA receptors and induced long-term potential (LTP) production (Lu et al., 1997; Pape and Pare, 2010). This is considered a key process leading to the emotional valence of the CS (Fanselow and LeDoux, 1999; Mayford et al., 2012), indicating the important role of the amygdala glutamatergic system in the formation of conditioned fear memory. However, whether and how this system participates in the fear memory generalization remains to be defined.

Metabotropic glutamate receptor 5 (mGluR5) is a member of both metabotropic glutamate receptor Group I and G-protein-coupled receptors (GPCR) family. mGluR5 is widely distributed in the brain and is expressed on the post-synaptic and extra-synaptic membranes of neurons. This receptor tends to cluster around NMDARs and rapidly facilitates NMDAR-dependent LTP production (Lujan et al., 1996; Chen et al., 2017), which may relate to the mechanism of fear memory formation. The function of mGluR5 is largely dependent on its coupling protein, Gα_*q/11*_, which initiates a cascade reaction by activating protein lipase C (PLC). PLC hydrolyzes phosphatidylinositol and generate the second messengers to induce activation of protein kinase C (PKC) and the release of cytosolic calcium pool (Wang et al., 2020). At the same time, mGluR5 has several sites that can be phosphorylated by PKC (Alaluf et al., 1995; Ciruela et al., 1999). Phosphorylation of mGluR5 blocks the coupling to the G protein, preventing the signal from being transmitted downstream (Minakami et al., 1997; Dhami and Ferguson, 2006). In addition to the neuronal cell membrane, there is another part of mGluR5 distributing in intracellular membrane structures such as the endoplasmic reticulum and nuclear membrane (Jong et al., 2014). These receptors play a role through G proteins or specific downstream signaling molecules as well.

Several studies have shown that mGluR5 plays a key role in formation and extinction of conditioned fear memory. It was reported that systematic administration of benzamide (3-cyano-N-(1,3-diphenyl-1H-pyrazol-5-yl) benzamide, CPDDB), a positive mutagen of mGluR5, promoted the formation and extinction of conditioned contextual fear memory (Hayashi, 2018). And other studies have found that systemic inhibition of mGluR5 could impair the formation of conditioned fear memory (Schulz et al., 2001; Shallcross et al., 2021). Studies on mGluR5 in the limbic system further elucidated these functions. Activation of mGluR5 in the lateral amygdala (LA) could facilitate the formation of conditioned fear memory (Rahman et al., 2017). Activation or inhibition of mGluR5 in the infralimbic amygdala (IL) can promote or inhibit the consolidation of extinction memory, respectively (Xu et al., 2009; Fontanez-Nuin et al., 2011). It is also reported that activation of mGluR5 in hippocampus would enhance contextual fear while delayed application of mGluR5 agonist eliminated fear enhancement (Tronson et al., 2010). However, it is unclear whether mGluR5 plays a role in the generalization of conditioned fear memory. Therefore, in this study we aimed to determine whether and how mGluR5 in the amygdala is involved in fear memory generalization.

## 2. Materials and methods

### 2.1. Animals

All experiments were performed with 8- to 10-week-old male C57BL6 mice obtained from the laboratory animal center of Sun Yat-sen University. Mice were housed in cages of 3–5 mice each and were kept at the standard temperature (22 ± 3°C) and the cycle of light/darkness was 12/12 h with lights on at 08:00 am. Animal experiments were conducted in accordance with the “Guidelines for the Care and Use of Laboratory Animals” issued by the National Institutes of Health (NIH) and approved by the Ethics Committee of Sun Yat-sen University School of Medicine, Sun Yat-sen University. All behavioral procedures took place during the animal light cycle.

### 2.2. Surgery

Mice (22–28 g), anesthetized with sodium pentobarbital (10 mg/kg, i.p.), were mounted on a stereotaxic apparatus and cannulas (both were obtained from RWD Life Science) were implanted into the bilateral amygdala [anteroposterior, 1.4 mm; mediolateral, ±3.2 mm; dorsoventral, −4.5 mm]. A 26-gauge dummy cannula was inserted into each cannula to prevent clogging. The mice were monitored and handled daily and were given 7 days to recover after surgery.

For bilateral AAV injection, mice were anesthetized as described above. A 1 μm Hamilton microsyringe containing AAV solution was targeted to both hemispheres (100 nl of AAV solution per side) of the amygdala [anteroposterior, 1.4 mm; mediolateral, ±3.2 mm; dorsoventral, −4.9 mm]. A microsyringe pump and its controller were used to control the speed of injection. The microsyringe was slowly lowered to the target site and was left in place for an additional 5 min after injection to ensure diffusion. The AAV solutions were injected at a constant rate of 20 nl/min. After bilateral injection the mice were placed in a warm room until awakening. After 14 days recovery, these mice were handled for 7 days continuously before behavior experiments.

### 2.3. Behavior

#### 2.3.1. Cued fear conditioning (CFC)

For CFC training, a square metallic chamber with an electric grid floor was used as context A. Mice were presented in context A and allowed free exploration for 180 s. After exploration, 1 and 5 kHz tones were delivered pseudo-randomly with an average interval of 70 s for 5 trials. Then 1 and 5 kHz were delivered pseudo-randomly for 10 trials with or without 1 s foot shock co-terminated, respectively. 1 kHz tone pair with foot shock was served as the conditioned danger cue (CS+, 75 dB, 10 s) and 5 kHz was served as conditioned safety cue (CS-, 75 dB, 10 s, pulse-width of 0.5 s).

For the fear memory retrieval test, 24 h after CFC training mice were presented in context B, a context-shifted chamber with glossy walls colored by red and white transverse stripes and a flat floor. Following 180 s free exploration, the above-mentioned tones were delivered pseudo-randomly for 5 trials.

**FIGURE 1:**
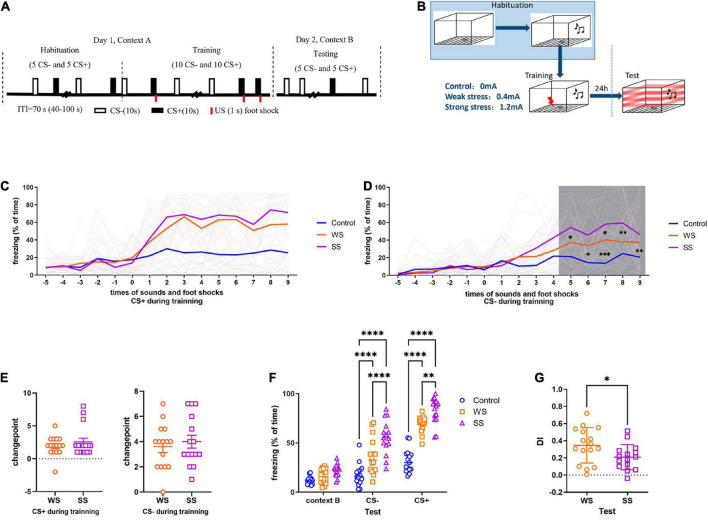
Fear conditioning by foot shock of different intensities caused incremental generalization. **(A,B)** Fear conditioning paradigm (15 mice in each group). **(C)** On day 1, no significant difference was found in the freezing to CS+ between WS and SS groups (two-way ANOVA, *F*_2,43_ = 33.45, *p* < 0.0001, Tukey’s multiple comparisons test). **(D)** Mice in SS group had higher freezing to CS- than that in WS group (two-way ANOVA, *F*_2,43_ = 7.377, *p* = 0.0018, Tukey’s multiple comparisons test). *p_5[Control vs SS]_ = 0.0131, *p_6[Control vs SS]_ = 0.0125, ^**^p_7[Control vs WS]_ = 0.0022, ^***^p_7[Control vs SS]_ = 0.0001, ^**^p_8[Control vs SS]_ = 0.0066, *p_9[Control vs SS]_ = 0.0227. **(E)** No significant difference was found in changepoint between WS and SS groups. **(F)** On day 2, SS group had significantly higher freezing than control and WS group (two-way ANOVA, *F*_2,43_ = 65.87, *p* < 0.0001. Tukey’s multiple comparisons test, CS- ^****^p_[Control vs WS]_ < 0.0001, ^****^p_[Control vs SS]_ < 0.0001, ^****^p_[WS vs SS]_ < 0.0001, CS+ ^****^p_[Control vs WS]_ < 0.0001, ^****^p_[Control vs SS]_ < 0.0001, ^****^p_[WS vs SS]_ = 0.0045). **(G)** Mice in SS group had significantly lower DI (unpaired two-tailed Student *t*-test, Two-tailed, *t* = 2.129, df = 29, *p = 0.0418).

The foot shock intensity included 0, 0.4, and 1.2 mA, which were marked as Control, weak-shock (WS), and strong-shock (SS), respectively (Figures 1A, B). To avoid odor interference, 75% ethanol and 4% acetic acid solution were used as a background odor during training and test, respectively. The activity of the mice in the chamber was recorded by the FreezeFrame system (Actimetrics Inc., Wilmette, Evanston, IL, USA). The average percentage of freezing (defined as immobility) during tones delivered was calculated. Changepoint detection was applied to analyze the CFC training data. A changepoint was defined as a certain statistical feature (distribution type and distribution parameter) that changed at a certain time point under the influence of systemic factors rather than accidental factors in a sequence or process. In the present study, we detected the changepoints of freezing during CFC training to determine after how many foot shocks the freezing to both CS+ and CS- of mice increased systematically (Killick and Eckley, 2014). So that we can infer the time points when these mice establish the basic fear reflex.

The discrimination index (DI) was calculated for evaluating the level of fear generalization (Xu and Südhof, 2013; Hao et al., 2023). For individual animal, it was defined as:


$$D\,I=\sum_{i=1}^{N_{t\,r\,i\,a\,l}}\left(\frac{F\,z_{i}^{C\,S+}-F\,z_{i}^{C\,S-}}{F\,z_{i}^{C\,S+}+F\,z_{i}^{C\,S-}}\right)/N_{t\,r\,i\,a\,l}$$

#### 2.3.2. Open field test (OFT)

The open field tests were processed in a top-opened acrylic chamber (50 cm × 50 cm), which was placed in a soundproof box. The mouse was placed in the center of the chamber at the beginning of the OFT. The activity trace was recorded for 5 min with an overhead video-tracking system (Shanghai Jiliang Software Technology Co., Ltd). The time mice spent in the center area, the total distance, and the velocity were calculated and analyzed.

### 2.4. Drug infusion

2-methyl-6-phenylethynyl-pyridine(MPEP, CAS: 96206-92-7, Aladdin^®^) was dissolved in saline at 0.016 mg/ml. Cannula dummies were removed from guide cannulas and replaced with 26-gauge injectors, which were connected by polyethylene tubing (RWD Life) to 1000-ul microliter syringes mounted in a CMA 402 Microdialysis Syringe Pump. Saline or MPEP was infused 30 min before CFC training at a rate of 0.1 μl/min for 2 min. Cycloheximide (CHX) was dissolved in saline at 50 μg/μl (Quadagno, 1976). 200 nl solution or saline was injected as described above.

### 2.5. Western blotting

The amygdala was dissected and frozen in liquid nitrogen immediately and stored at −80°C for later analysis. Synaptic and cytosolic fractions were obtained as described by Holz et al. (2019). In brief, all tissues were mechanically homogenized with homogenization buffer (320 mM sucrose, 4 mM HEPES [pH 7.4], 2 mM EDTA) and centrifuged at 800 g for 10 min at 4°C to generate total (S1) and nuclear fraction (P1). Synaptic (P2) and cytosolic (S2) fractions were obtained by centrifugation of S1 at 10000 g for 20 min at 4°C. The synaptic fraction was lysed in lysis buffer (50 mM Tri-HCl [pH 6.8], 1.3% SDS, 6.5% glycerol, 100 μM sodium orthovanadate). A phosphatase and protease inhibitor cocktail (Sigma-Aldrich, 100×) was added into those buffers just before use.

The protein concentration was measured by spectrophotometry at 580 nm using an absorbance reader (Elx800, BioTek Instrument, Inc). All the samples were heated at 65°C for 10 min before being loaded onto 4–12% Bis-Tris gels (GenScript) in the running buffer (GenScript) and separated at 80°CV for 2 h on ice. Activated PVDF membrane with methanol for 10 min and rinse with transfer buffer before preparing the stack. The proteins were then transferred to a polyvinylidene fluoride membrane (ISEQ00010, Millipore) at 70 V for 30 min using a Tanon trans blot system. Membranes were then blocked using 5% Difco™ Skim Milk (232100, BD) in 0.1% TBST (TBS-Tween 20) for 2 h at room temperature and incubated with the mGluR5 (ab76316, Abcam, 1:1000) and GAPDH (2118S, CST, 1:6000) primary antibody overnight at 4°C. Membranes were washed with 0.1% TBST 3 times for 10 min each, incubated with the secondary antibody (7074S, CST) for 1 h at room temperature, and subsequently washed 3 times for 5 min each. For signal detection, the membranes were incubated with Pierce ECL Western Blotting Substrate (WBULS0100, MERCK) for 5 min and then imaged using a GeneGnome XRQ Chemiluminescence Imaging System.

### 2.6. RNA extraction and quantitative polymerase chain reaction (qPCR)

The amygdala of conditioned mice was separated immediately after CFC training. The total RNA was extracted and purified using HP Total RNA Kit (R6812-01 OMEGA bio-tek). Reverse transcription was conducted with the Color reverse transcription kit (A0010CGQ EZBionscience, USA) and qPCR was performed by 2 × color SYBR Green qPCR Master Mix (A0012-R2ROX2 plus EZBionscience, USA). Primer sequences are listed in Supplementary Table 1.

### 2.7. Immunohistochemistry and imaging analysis

To evaluate the activity of the neurons in the amygdala, the cfos expression on neurons was calculated in the amygdala. Mice were sacrificed 90 min after fear conditioning. Brian slices (thickness of each slice was 40 μm) separated by 240 μm were immunostained with rabbit anti c-Fos antibody (1:500, 2250S, CST) followed by Alexa Flour 555 conjugated anti-rabbit IgG (1:2000, 4413S, CST) and DAPI (1:5000, CAS: 28718-90-3, Sigma^®^). Images were acquired using Leica upright fluorescence microscope (DM6B) using 20× objective and a pixel size of 72 nm. The boundaries of subregions of the amygdala were based on the mouse brain atlas. The c-Fos^+^ neurons were counted by ImageJ software.

### 2.8. Statistical analysis

The thresholds for significance were placed at *p < 0.05, ^**^p < 0.01, and ^***^p < 0.001. All data were shown as mean and SEM unless stated otherwise. Unpaired Student’s *t*-test, two-way ANOVA, and one-way ANOVA followed by a Turkey’s multiple comparison or a Fisher’s LSD test were performed using GraphPad Prism 9.

## 3. Results

### 3.1. Fear conditioning with increasing foot shock intensity caused incremental fear generalization

To address how foot shock contributes to cued fear memory, different intensities of foot shock were delivered to mice. During CFC training, there was no significant difference between the WS and SS groups compared to the control group in freezing to CS+, although the freezing level was higher in both WS and SS groups (Figure 1C). As to CS-, both WS and SS groups showed significantly higher freezing levels compared to the control, especially in the last 5 CS- trials (Figure 1D). Changepoint analysis showed that neither to CS+ nor CS-, there was no significant difference between the WS and SS group compared to the control group in changepoint (Figure 1E). During the test, both WS and SS groups showed significantly higher freezing levels of both CS+ and CS- compared to the control group (Figure 1F). Although, the SS group had significantly higher freezing levels of CS+ and CS- compared to the WS group (Figure 1F). The DI of the SS group was significantly lower compared to the WS group (Figure 1G). These results indicating due to the intensities increase of foot shock stress, fear memory of threat cue (CS+) was generalized to safe cue (CS-). It suggested that the ability to distinguish between threat and safe was damaged by severe stress stimuli, and the time point when the damage started differs during stress happened might be the determination of the extent of fear memory generalization.

### 3.2. Density of c-Fos^+^ cells failed to distinguish the intensity of stress

**FIGURE 2:**
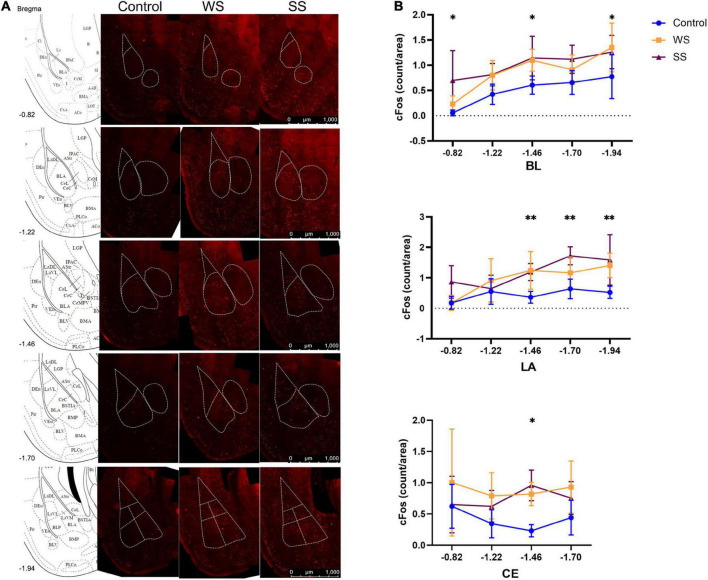
c-Fos^+^ cells in amygdala after fear conditioning. **(A)** c-Fos immunofluorescent in the amygdala (4 mice in each group). **(B)** c-Fos^+^ cells density in amygdala increased after fear conditioning (two-way ANOVA, Tukey’s multiple comparisons test, BL: *F*_2,9_ = 18.34, *p* < 0.0001, *p_[–0.82, Control vs SS]_ = 0.0275, *p_[–1.46, control vs WS]_ = 0.0491, *p_[–1.46, control vs SS]_ = 0.0115, *p_[–1.94, control vs WS]_ = 0.0221; LA: *F*_2,56_ = 18.45, *p* < 0.0001, ^**^p_[–1.46, control vs WS]_ = 0.0053, ^**^p_[–1.46, control vs SS]_ = 0.0028, ^**^p_[–1.70, control vs SS]_ = 0.0027, ^**^p_[–1.94, control vs WS]_ = 0.0069, ^**^p_[–1.94, control vs SS]_ = 0.0010; CE: *F*_2,45_ = 10.42, ^**^p = 0.0002, *p*_[–1.46, control vs WS]_ = 0.0073, ^***^p_[–1.46, control vs SS]_ = 0.0002).

To determine the functional response of the amygdala during cued fear memory acquisition, we observed the neuronal activation in the LA, basal lateral nucleus of the basal lateral amygdala (BL), and central nucleus of the amygdala (CE) during training by immunostaining of neuronal activity marker c-Fos (Figure 2A). Imaging analysis showed that compared with the control group, the density of the c-Fos^+^ cell population was significantly higher in both WS and SS mice in the BL. Such an increase was also observed in posterior LA. No significant difference was found in the CE except one layer. However, there was no significant difference between the WS and SS group in the three subregions of the amygdala (Figure 2B). These results indicated that CFC training caused the amygdala activation, but the activation level did not increase with the enhancement of foot shock intensity.

### 3.3. Chemo-genetic inhibition of amygdala impaired the level of fear memory rather than generalization

**FIGURE 3:**
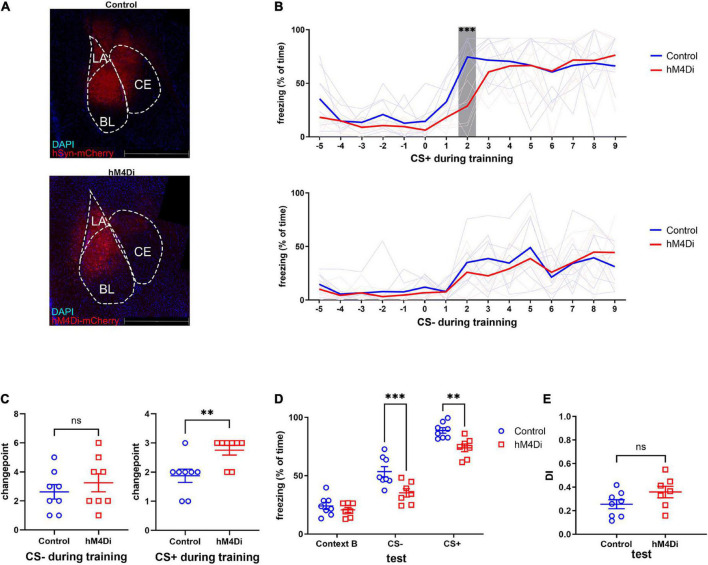
Chemo-genetic inhibition of amygdala impaired fear memory rather than fear generalization. **(A)** Images of hM4Di and control virus expression in the amygdala (red: mCherry, blue: DAPI). **(B)** Freezing to CS+ decreased under amygdala inhibition (two-way ANOVA, *F*_1,14_ = 2.25, *p* = 0.1558, Tukey’s multiple comparisons test, ^****^p_2[CS+, CTR vs hM4Di]_ < 0.0001). Freezing to CS- was not affected (two-way ANOVA, *F*_1,14_ = 0.3796, *p* = 0.5477). **(C)** Inhibition of amygdala caused a delay on changepoint of fear response to CS+ during training (CS+ unpaired two-tailed Student *t*-test, Two-tailed, *t* = 3.130, df = 14, ^**^p = 0.0074), but did not influence the changepoint of fear response to CS- (CS- unpaired two-tailed Student *t*-test, Two-tailed, *t* = 0.7864, df = 14, *p* = 0.4448). **(D,E)** Test after 24 h, amygdala inhibition caused fear memory impairment, but DI was not affected (test, two-way ANOVA, *F*_1,13_ = 13.64, *p* = 0.002, ^***^p_[cs–]_ = 0.0008, ^**^p_[cs+]_ = 0.007. DI, unpaired two-tailed Student *t*-test, Two-tailed, *t* = 1.693, df = 13, *p* = 0.1143).

To further explore the relationship between activation of the amygdala and fear memory generalization, we expressed the human M4 muscarinic (hM4) receptor in the amygdala (Figure 3A). Clozapine N-oxide (CNO) was injected intraperitoneally 30 min before a SS CFC training. There was no difference in freezing to CS-, but the freezing to the second CS+ block of the control group was significantly higher than that of the hM4Di group (Figure 3B). Changepoint analysis showed that mice in the hM4Di group had a delayed fear response to CS+, indicating that mice needed more shocks before reaching the highest fear response when the BLA was inhibited (Figure 3C). At 24 h after conditioning, the cued fear memory was measured as described above. Freezing responses to CS- and CS+ of the hM4Di group were both significantly lower compared to the control group (Figure 3D), while the DI remained unaltered (Figure 3E). Meanwhile, the open-field test showed locomotory ability and anxiety level of the hM4Di group were not significantly different from those of the control group (Supplementary Figure 1). The results above suggested that the overall activation level of the amygdala is not related to the extent of fear memory generalization but the intensity of fear memory, and this alteration in intensity is not due to anxiety.

### 3.4. mGluR5 expression alteration caused by fear conditioning was time- and stress intensity- dependent

We tested whether mGluR5 was modulated by foot shock stress and whether this modulation was affected by shock intensities. To start with, CFC training was performed as above. The amygdala was isolated bilaterally at 0, 8, or 24 h after training, and then P2 and S2 fractions were obtained through differential centrifuge as described above.

**FIGURE 4:**
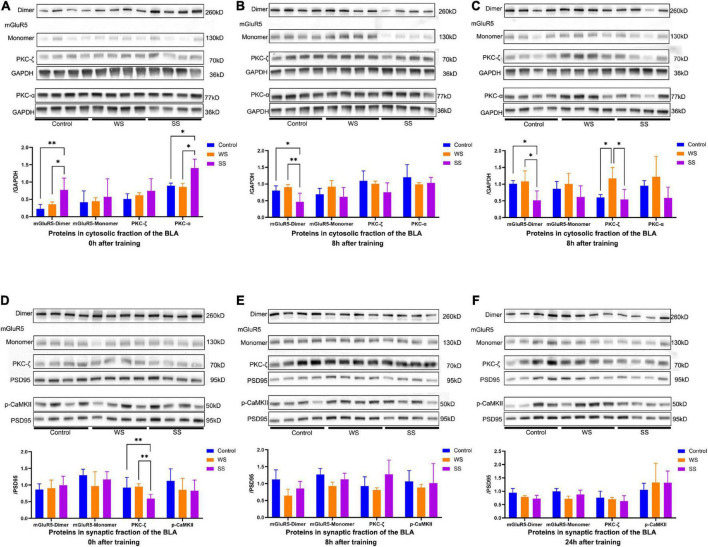
mGluR5-dimer and PKC-α expression in the amygdala of mice at different time point after fear conditioning. **(A)** mGluR5-dimer and PKC-α expressions were increased in the cytosolic fraction of amygdala from SS group in immediately after fear conditioning but S2 mGluR5-monomer and PKC-ζ expressions were not affected by fear conditioning (0 h, one-way ANOVA, mGluR5-dimer, *F*_2,9_ = 6.988, *p* = 0.0147, multiple comparison, Fisher’s LSD test, *p*_[Control vs WS]_ = 0.3991, ^**^p_[Control vs SS]_ = 0.0059, *p_[WS vs SS]_ = 0.0243; mGluR5-monomer, *F*_2,9_ = 0.2094, *p* = 0.8149; PKC-ζ, *F*_2,9_ = 1.072, *p* = 0.3824; PKC-α, *F*_2,9_ = 14.17, *p* = 0.0017, multiple comparison, Fisher’s LSD test, *p*_[Control vs WS]_ = 0.7988, *p_[Control vs SS]_ = 0.015, *p_[WS vs SS]_ = 0.0011). **(B,C)** mGluR5-dimer expression was decreased in the S2 fraction of the amygdala from SS group 8 h and 24 h after fear conditioning but S2 mGluR5-monomer, PKC-ζ and PKC-α expressions in WS and SS group were consist with control group **(B)**, 8 h, one-way ANOVA, mGluR5-dimer, *F*_2,9_ = 6.823, *p* = 0.0157, multiple comparison, Fisher’s LSD test, *p*_[Control vs SS]_ = 0.4347, *p_[Control vs SS]_ = 0.024, ^**^p_[WS vs SS]_ = 0.0064; mGluR5-monomer, *F*_2,9_ = 2.121, *p* = 0.1759; PKC-ζ, *F*_2,9_ = 2.151, *p* = 0.1723; PKC-α, *F*_2,9_ = 0.8745, *p* = 0.4497. **(C)** 24 h, one-way ANOVA, mGluR5-dimer, *F*_2,9_ = 6.114, *p* = 0.0210, multiple comparison, Fisher’s LSD test, *p*_[Control vs WS]_ = 0.6839, *p_[Control vs SS]_ = 0.0208, *p_[WS vs SS]_ = 0.0105; mGluR5-monomer, *F*_2,9_ = 1.788, *p* = 0.2219; PKC-ζ, *F*_2,9_ = 7.298, *p* = 0.0131, multiple comparison, Fisher’s LSD test, *p_[Control vs WS]_ = 0.012, *p_[Control vs SS]_ = 0.7527, *p_[WS vs SS]_ = 0.0072, PKC-α, *F*_2,9_ = 2.417, *p* = 0.1444). **(D)** PKC-ζ expression was deceased in the P2 fraction of amygdala from SS group immediately after fear conditioning but P2 mGluR5 and p-CaMKII expressions were not altered (0 h, one-way ANOVA, mGluR5-dimer, *F*_2,9_ = 0.3339, *p* = 0.7247; mGluR5-monomer, *F*_2,9_ = 1.209, *p* = 0.3427; PKC-ζ, *F*_2,9_ = 4.058, *p* = 0.0554, multiple comparison, Fisher’s LSD test, *p*_[Control vs WS]_ = 0.8331, ^**^p_[Control vs SS]_ = 0.0432, ^**^p_[WS vs SS]_ = 0.0303; p-CaMKII, *F*_2,9_ = 0.8984, *p* = 0.4408). **(E,F)** No significant changes of synaptic mGluR5, PKC-ζ and p-CaMKII were detected in the P2 fraction of amygdala 8 h, and 24 h after fear conditioning **(E)**, 8 h, one-way ANOVA, mGluR5-dimer, *F*_2,9_ = 3.34, *p* = 0.0822; mGluR5-monomer, *F*_2,9_ = 3.966, *p* = 0.0582; PKC-ζ, *F*_2,9_ = 2.6, *p* = 0.1209; p-CaMKII, *F*_2,9_ = 0.2292, *p* = 0.7997. **(F)** 24 h, one-way ANOVA, mGluR5-dimer, *F*_2,9_ = 3.719, *p* = 0.0665; mGluR5-monomer, *F*_2,9_ = 3.53, *p* = 0.0738; PKC-ζ, *F*_2,9_ = 0.4826, *p* = 0.6323; p-CaMKII, *F*_2,9_ = 0.3724, *p* = 0.6992).

Western blotting showed that after SS fear conditioning, the level of S2 mGluR5 dimer immediately increased significantly compared to the other groups. At the same time, we found that PKC-α (a downstream protein of mGluR5) showed similar expression patterns (Figure 4A). At 8 h after fear conditioning, however, the level of S2 mGluR5 dimer in the SS group showed a significant decrease compared with the other two groups (Figure 4B). This phenomenon continued until 24 h after fear conditioning (Figure 4C), but S2 PKC-α protein expression decreased to the normal level in the SS group after 8 h.

Although multiple studies on mGluR5 are based on the hypothesis that the synaptic part of this receptor is a major participator of various neuronal processes. We found that after fear conditioning, the expression of P2 mGluR5 had no significant alteration (Figures 4D–F). Given the mGluR5 might alter neuronal synaptic plasticity by regulating NMDA receptor phosphorylation levels (Chen et al., 2017), we further examined the expression levels of p-NMDA (p-GluN2) receptor and glutamine synthetase (GS) in the synaptic part. The P2 p-GluN2 expression in the amygdala of the SS group did not decrease until 8 h after training, and then remained at a relatively low level. Meanwhile, there was not significant difference in GS expressions in the amygdala P2 fractions was detected among the groups at different time points after fear conditioning (Supplementary Figure 2).

These results indicated that S2 mGluR5 protein increased rapidly, but then continuously decreased after SS CFC training, while WS did not cause significant changes in the expression of mGluR5 and its downstream protein. The expression of p-NMDAR showed a delayed alteration after training without the ability to distinguish the impact of shock intensity.

### 3.5. Increase of mGluR5 expression was *de novo* protein synthesis

**FIGURE 5:**
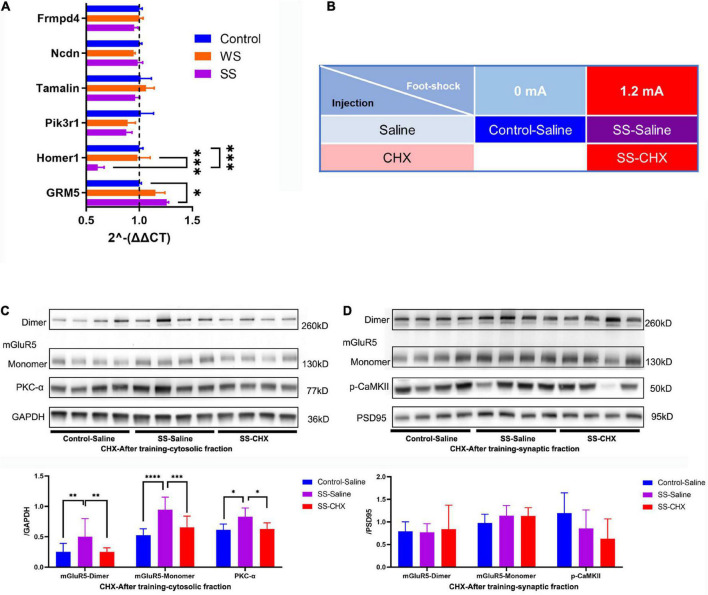
Increased expression of mGluR5 come from *de novo* protein synthesis. **(A)** RT-qPCR analysis of the GRM5, Homer1, Pik3r1, Tamalin, Ncdn and Frmpd4 in the amygdala of mice immediately after CFC training (4 mice in each group, two-way ANOVA, *F*_10,48_ = 3.265, *p* = 0.0027, Tukey’s multiple comparisons test, GRM5, *p*_[Control vs WS]_ = 0.2473, *p_[Control vs SS]_ = 0.0225, *p*_[WS vs SS]_ = 0.4480; Homer1, *p*_[Control vs WS]_ = 0.9813, ^***^p_[Control vs SS]_ = 0.0003, ^***^p_[WS vs SS]_ = 0.0002; Pik3r1, *p*_[Control vs WS]_ = 0.3920, *p*_[Control vs SS]_ = 0.3287, *p*_[WS vs SS]_ = 0.9905; Tamalin, *p*_[Control vs WS]_ = 0.8465, *p*_[Control vs SS]_ = 0.8618, *p*_[WS vs SS]_ = 0.4843; Ncdn, *p*_[Control vs WS]_ = 0.8415, *p*_[Control vs SS]_ = 0.9851, *p*_[WS vs SS]_ = 0.9045; Frmpd4, *p*_[Control vs WS]_ > 0.9999, *p*_[Control vs SS]_ = 0.8589, *p*_[WS vs SS]_ = 0.8431. **(B)** Injection paradigms for all groups (*N* = 4 for every group). **(C)** CHX impaired the increase of S2 mGluR5 and PKC-α expressions in the amygdala after CFC training (4 mice in each group, one-way ANOVA, mGluR5-dimer, *F*_2,9_ = 5.564, *p* = 0.0267, multiple comparison, Fisher’s LSD test, *p_[Control–Saline vs SS–Saline]_ = 0.0135, *p*_[Control–Saline vs SS–CHX]_ = 0.7108, *p_[SS–Saline vs SS–CHX]_ = 0.0253; mGluR5-monomer, *F*_2,9_ = 6.624, *p* = 0.0170, multiple comparison, Fisher’s LSD test, ^**^p_[Control–Saline vs SS–Saline]_ = 0.0061, *p*_[Control–Saline vs SS–CHX]_ = 0.2772, *p_[SS–Saline vs SS–CHX]_ = 0.0392; PKC-α, *F*_2,9_ = 8.855, *p* = 0.0075, multiple comparison, Fisher’s LSD test, ^**^p_[Control–Saline vs SS–Saline]_ = 0.01, *p*_[Control–Saline vs SS–CHX]_ = 0.5079, ^**^p_[SS–Saline vs SS–CHX]_ = 0.0034). **(D)** mGluR5 and PKC-α expressions in BLA P2 fraction were not changed by SS CFC training and CHX injection (4 mice in each group, one-way ANOVA, mGluR5-dimer, *F*_2,9_ = 0.04079, *p* = 0.9602; mGluR5-monomer, *F*_2,9_ = 0.8303, *p* = 0.4667, p-CaMKII *F*_2,9_ = 1.729, *p* = 2315).

To determine the mechanism in cytosolic mGluR5 protein increase, we examined the mRNA expression of GRM5 and its relative genes including Frmpd4, Ncdn, Tamalin, Pik3r1, and Homer1 using reverse transcriptase quantitative polymerase chain reaction. Results showed that SS CFC training caused an enhancement in GRM5 mRNA expression and inhibited Homer1, a mGluR5 binding protein, transcription in the amygdala. However, we failed to detect a significant difference in GRM5 mRNA expression between the WS and SS group, indicating there might be GRM5 expression enhancement below the statistical threshold after WS fear conditioning. The mRNA expression of the remaining genes remained basically unchanged (Figure 5A).

Given the transcription enhancement of GRM5 after SS fear conditioning, we hypothesized that the increase of S2 mGluR5 protein is due to *de novo* protein synthesis. To prove this hypothesis, mice were randomly divided into three groups. Saline was injected into the mice (labeled as control-saline group) that did not receive foot shock. Then, CHX-saline solution or same-volume saline was injected to the SS group, respectively (labeled as SS-CHX or SS-saline) (Figure 5B). We found that increase of mGluR5 and PCK-α caused by SS fear conditioning was not interfered with cannulas implant and saline injection. Meanwhile, CHX injection significantly inhibited the increase of mGluR5 and PKC-α expression, compared with saline injection (Figures 5C, D). These data suggested there was a *de novo* mGluR5 synthesis in the amygdala during fear conditioning.

### 3.6. Inhibition of mGluR5 during fear conditioning has a stress intensity dependent effect on fear memory generalization

**FIGURE 6:**
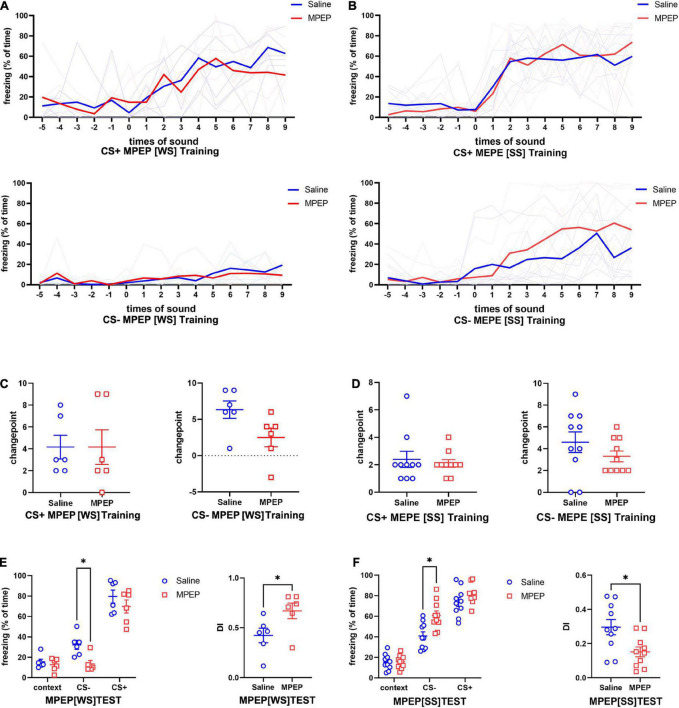
Inhibition of mGluR5 during fear conditioning leaded to a stress intensity dependent alteration on fear memory generalization. **(A,B)** Significant differences were not found in fear response to CS+ or CS- between control group and MPEP group regardless of the given foot shock intensity **(A)**, two-way ANOVA CS+[WS] *F*_1,10_ = 0.0004339, *p* = 0.9838; CS-[WS] *F*_1,10_ = 1.050, *p* = 0.3297; **(B)** two-way ANOVA CS+[SS] *F_1,18_* = 0.01473, *p* = 0.9047; CS-[SS] *F_1,18_* = 1.273, *p* = 0.2741). **(C)** Changepoints on freezing to CS+ or CS- were not altered by MPEP treatment during WS CFC training (CS+[WS], unpaired two-tailed Student *t*-test, Two-tailed, *t* = 0.000, *df* = 10, *p* > 0.9999; CS-[WS], unpaired two-tailed Student *t*-test, Two-tailed, *t* = 2.157, *df* = 9, *p* = 0.0594). **(D)** The changepoints to CS- rather than CS+ in the MPEP group were significant lower than the saline group during SS CFC training (CS+[SS], unpaired two-tailed Student *t*-test, Two-tailed, *t* = 0.4660, *df* = 18, *p* = 0.6468; CS-[SS], unpaired two-tailed Student *t*-test, Two-tailed, *t* = 1.218, *df* = 18 *p* = 0.2389). **(E)** MPEP leaded to decreased fear response to CS- and increased DI during test when using WS foot shock training (two-way ANOVA, *F*_1,10_ = 8.860, *p* = 0.0139, Tukey’s multiple comparisons test, *p_[CS–]_ = 0.0221, *p*_[CS+]_ = 0.9691; DI, unpaired two-tailed Student *t*-test, Two-tailed, *t* = 2.305, *df* = 10, *p = 0.0439). **(F)** MPEP treatment combined with SS foot shock in training causes increased fear response to CS- and decreased DI during test (two-way ANOVA, *F*_1,18_ = 7.577, *p* = 0.0131, Bonferroni’s multiple comparisons test, *p_[CS–]_ = 0.0102, *p*_[CS+]_ = 0.5044; DI, unpaired two-tailed Student *t*-test, Two-tailed, *t* = 2.720, *df* = 18, *p = 0.0141).

Based on the above data, we assumed that mGluR5 might take a key part in fear generalization. To further explore the functional relationship between mGluR5 and fear memory generalization, we implanted cannulas into the bilateral amygdala of mice again (Supplementary Figure 3A). MPEP/saline solution or saline was injected into the amygdala 30 min before fear conditioning. The open-field test showed locomotory ability and anxiety level of the MPEP group were not significantly different from those of the control group (Supplementary Figures 3B, C). No significant difference was found in freezing to CS- or CS+ (Figures 6A, B) and changepoint (Figures 6C, D) between the MPEP group and saline group during CFC training, regardless of SS or WS. During the test, MPEP did not change the fear response of mice to CS+ regardless of SS or WS. However, MPEP injection could significantly diminish fear response to CS- with an increase in DI after WS CFC training, indicating fear generalization level was decreased (Figure 6E). When SS was presented as unconditional stress, the fear response to CS- of the MPEP group was contrarily increased with decreased DI, suggesting that fear generalization was enhanced (Figure 6F).

## 4. Discussion

The fear response of animals during the training has been regarded as the fear effect induced by acute stress (Wiltgen and Silva, 2007; Zhou et al., 2017). However, our study showed that during acquisition, after the presence of stress, the animals rapidly developed a freezing response to the acoustic stimulus (CS+) paired with foot shock. This response is different from the avoidance response of animals running away produced when they are exposed to electric stimuli (US), such as jumping and escaping. It resembles the freezing response exhibited during the retrieval test, indicating that at this time the classic conditioned fear memory reflex has been initially established (LeDoux, 2014). Meanwhile, the fear responses to unpaired sound stimuli (CS-) are also gradually established during the training process. The peak fear responses among the control, WS, and SS group are significantly different, and are generally consistent with the pattern of the mice’s response to the two sound stimuli during retrieval. Generalization has long been regarded as memory inaccuracy or a mismatch based on information processing and comparison during consolidation (Poldrack and Packard, 2003; Zaki et al., 2003; Poldrack and Foerde, 2008). But our results suggested that the fear generalization and the formation of conditioned fear memory could begin simultaneously from the acquisition, rather than from the consolidation stage. Even in the acquisition process, the level of fear generalization has been determined to some extent.

Based on the dual pathway hypothesis of fear learning and expression, the amygdala is central to the conditioned fear response (Lissek et al., 2010). So, we tried to investigate the relationship between the amygdala activation levels during the fear conditioning and the level of fear memory generalization. Ghosh and Chattarji have reported the so-called “generalized cells” during retrieval, whose responses to CS- and CS+ are different. They thought that the process of generalization was a transformation of “specific cells” into “generalized cells” without new cells recruitment. In this study, we found the density of c-Fos^+^ cell did not change following stress enhancement, which somehow proves Ghosh’s opinion. Then we attempted to inhibit this transformation process with chemical genetic techniques. Under strong foot shock, inhibition of the amygdala significantly impaired conditioned fear memory but had no significant effect on the level of fear memory generalization. These results indicate that robustly inhibiting the amygdala cannot prevent the transformation of “specific cells” into “generalized cells” but impair the ability to form conditioned fear responses.

Previous studies have shown that memory formation requires two phases of protein synthesis, an initial phase that begins during or after training, and a second phase that begins 3–6 h after training. The formation of multiple memories such as olfactory recognition memory and social fear memory can be impaired due to the inhibition of early *de novo* protein synthesis by administering a protein synthesis blocker 20–30 min before training (Bourtchouladze et al., 1998). Given the density of c-Fos^+^ cells in the amygdala after training does not change significantly in response to changes in stress intensity, we speculate that there may be alterations in the expression characteristics of certain molecules in the amygdala during training, which is responsible for “recording” the intensity of stress, influencing or even determining the level of conditioned fear generalization, since during consolidation these mice are confined in home-cage and cannot receive stress intensity information again.

A growing body of evidence indicates that mGluR5 in the amygdala plays an important role in multiple stages of conditioned fear memory formation and retrieval. In the present study, we found that there were distinct molecular processes occurring in the cytoplasm and synapses of amygdala cells after fear conditioning. The expression level of cytosolic mGluR5 dimer increased rapidly after SS training, following a rapid decrease within a few hours. In contrast, the expression level of synaptic mGluR5 remained relatively stable after training. To further investigate the relationship between mGluR5 expression and synaptic plasticity, we examined the changes in p-NMDA receptor expression levels in synapses, and found that the decrease in the expression level of synaptic p-NMDA receptor at 8 and 24 h could not distinguish WS and SS.

There is a complex interaction between mGluR5 and NMDAR. Activation of mGluR5 promotes NMDAR-mediated response enhancement through the PKC signaling pathway. Although it does not directly induce NMDAR-dependent LTP production, it can lower the threshold of LTP production and enhance neuronal excitability (Chen et al., 2011). The activation of NMDA receptors phosphorylates mGluR5 through the PKC signaling pathway. Phosphorylation then reduces themGluR5 functional level, forming a feedback regulation (Alagarsamy et al., 2002). The qPCR result demonstrated a significant enhancement in GRM5 transcription in the amygdala following SS training, which was generally consistent with the western blotting results. The CHX injection significantly inhibited mGluR5 dimers and monomers, and then increased PKC-α by SS fear conditioning, indicating that these proteins are synthesized *de novo* during the training. We think that after fear conditioning, neurons in the amygdala will rapidly synthesize mGluR5, and then its expression level will be continuously suppressed by the negative feedback mechanism and kept at a lower level (Francesconi and Duvoisin, 2000; Dhami and Ferguson, 2006). Interestingly, the results from Jong et al. (2014) support that mGluR5 on the endoplasmic reticulum and nuclear membrane surface can perform its specific functions through intracellular signaling pathways. These results suggest that the amygdala may “record” the individual stress magnitude through intracellular expression level of mGluR5 during fear conditioning, thereby determining the extent of specific or generalized conditioned fear memory.

Given the level of NMDAR phosphorylation did not change with stress intensities and generalization levels of mice, we further investigated the role of mGluR5 in cued fear memory generalization by intracranial injection of MPEP. Gold and Wrenn (2012) found that CHX only inhibited high-intensity stress-triggered conditioned fear, but CHX had no effect on low-intensity shock-triggered conditioned fear responses, suggesting that the stress-intensity-dependent modulation of conditioned fear memory may be related to the process of *de novo* protein synthesis. In the present study, the WS CFC did not increase the levels of mGluR5 expression during conditioned fear memory formation, while inhibition of mGluR5 with MPEP significantly inhibited generalization of conditioned fear memory, suggesting that mGluR5 promotes generalization of conditioned fear memory under physiological conditions. In contrast, after SS CFC, mGluR5 was rapidly synthesized, and the expression level of mGluR5 in the cytoplasm increased significantly, while the expression level of mGluR5 in the synaptic part did not, suggesting that the mGluR5 function on the synaptic surface may be in a state of inhibition and further inhibition of mGluR5 at this time would promote the generalization of conditioned fear memory in animals. Thus, it is hypothesized that the physiological function of mGluR5 is to promote the generalization of conditioned fear memory; during SS CFC training, mGluR5 function was inhibited and mGluR5 expression levels increased through a feedback mechanism, of which the mechanism needs further exploration.

Schulz et al. (2001) found that systematic administration of MPEP 60 min prior to conditioned fear memory training inhibited the formation of conditioned fear memory. But this study showed that the intracranial administration of MPEP prior to training did not affect the level of response to CS+ during training or retrieval test. MPEP may act on the amygdala and other brain regions such as the prefrontal cortex, which participate in the acquisition of conditioned fear memory. mGluR5 in the amygdala is not directly involved in regulating the formation of specific conditioned fear memory, and the inhibitory effect of MPEP on the formation of specific fear memory may be mediated by inhibiting mGluR5 in other brain regions.

In addition, it has been suggested that mGluR5 rapidly dimerizes after intracellular synthesis of monomers and is transferred intracellularly as a dimer (Parnot and Kobilka, 2004). However, Wang et al. (2020) observed the presence of mGluR5 monomers in adult brains. It has also been reported that mGluR5 undergoes aggregation and depolymerization in response to external signals *in vivo* (Harney et al., 2006; Sun et al., 2020). In our study, we observed mGluR5 monomers in both synaptic and cytoplasmic fractions of the amygdala by western blotting. Although the expression levels of mGluR5 monomers in the cytoplasm were not statistically significantly different between different groups at different stages after training, the trend of mGluR5 monomers in both cytoplasmic and synaptic fractions is basically the same as that of dimers, which supported the view of Parnot and Kobilka (2004) to a certain extent.

In summary, our study confirms that the level of fear generalization is determined during training. mGluR5 in the amygdala can serve as a molecular marker to define the stress intensity dependent fear generalization and play a significant role in the modulation of that. In the future, we will explore the mechanism of how mGluR5 regulates fear generalization in a stress intensity dependent manner. The findings of this study endorse our further understanding of the mechanism of PTSD and may propose new therapeutic strategies.

## Data availability statement

The data presented in this study are deposited in the figshare repository, doi:10.6084/m9.figshare.21638084.

## Ethics statement

The animal study was reviewed and approved by the Institutional Animal Care and Use Committee, Sun Yat-sen University.

## Author contributions

S-MX designed the research. S-MX and Y-WS performed the research, analyzed the data, and wrote the manuscript. All authors contributed to the article and approved the submitted version.

## References

[B1] Alagarsamy S. Rouse S. T. Junge C. Hubert G. W. Gutman D. Smith Y. (2002). NMDA-induced phosphorylation and regulation of mGluR5. *Pharmacol. Biochem. Behav.* 73 299–306. 10.1016/S0091-3057(02)00826-2 12117583

[B2] Alaluf S. Mulvihill E. McIlhinney R. (1995). Rapid agonist mediated phosphorylation of the metabotropic glutamate receptor 1 alpha by protein kinase C in permanently transfected BHK cells. *FEBS Lett.* 367 301–305. 10.1016/0014-5793(95)00575-T 7607328

[B3] Bourtchouladze R. Abel T. Berman N. Gordon R. Lapidus K. Kandel E. R. (1998). Different training procedures recruit either one or two critical periods for contextual memory consolidation, each of which requires protein synthesis and PKA. *Learn. Mem.* 5 365–374. 10.1101/lm.5.4.365 10454361 PMC311273

[B4] Chen A. Hu W. W. Jiang X. L. Potegal M. Li H. (2017). Molecular mechanisms of group I metabotropic glutamate receptor mediated LTP and LTD in basolateral amygdala in vitro. *Psychopharmacology* 234 681–694. 10.1007/s00213-016-4503-7 28028604

[B5] Chen H. H. Liao P. F. Chan M. H. (2011). MGluR5 positive modulators both potentiate activation and restore inhibition in NMDA receptors by PKC dependent pathway. *J. Biomed. Sci.* 18:19. 10.1186/1423-0127-18-19 21342491 PMC3050796

[B6] Ciruela F. Giacometti A. McIlhinney R. A. (1999). Functional regulation of metabotropic glutamate receptor type 1c: A role for phosphorylation in the desensitization of the receptor. *FEBS Lett.* 462 278–282. 10.1016/S0014-5793(99)01547-1 10622711

[B7] Compean E. Hamner M. (2019). Posttraumatic stress disorder with secondary psychotic features (PTSD-SP): Diagnostic and treatment challenges. *Prog. Neuro Psychopharmacol. Biol. Psychiatry* 88 265–275. 10.1016/j.pnpbp.2018.08.001 30092241 PMC6459196

[B8] Dhami G. K. Ferguson S. S. (2006). Regulation of metabotropic glutamate receptor signaling, desensitization and endocytosis. *Pharmacol. Ther.* 111 260–271. 10.1016/j.pharmthera.2005.01.008 16574233

[B9] Fanselow M. S. LeDoux J. E. (1999). Why we think plasticity underlying pavlovian fear conditioning occurs in the basolateral amygdala. *Neuron* 23 229–232. 10.1016/S0896-6273(00)80775-8 10399930

[B10] Fontanez-Nuin D. E. Santini E. Quirk G. J. Porter J. T. (2011). Memory for fear extinction requires mGluR5-mediated activation of infralimbic neurons. *Cereb. Cortex* 21 727–735. 10.1093/cercor/bhq147 20705895 PMC3041015

[B11] Francesconi A. Duvoisin R. M. (2000). Opposing effects of protein kinase C and protein kinase A on metabotropic glutamate receptor signaling: Selective desensitization of the inositol trisphosphate/Ca2+ pathway by phosphorylation of the receptor-G protein-coupling domain. *Proc. Natl. Acad. Sci. U.S.A.* 97 6185–6190. 10.1073/pnas.97.11.6185 10823959 PMC18579

[B12] Fulton J. J. Calhoun P. Wagner H. Schry A. Hair L. Feeling N. (2015). The prevalence of posttraumatic stress disorder in operation enduring freedom/operation iraqi freedom (OEF/OIF) veterans: A meta-analysis. *J. Anxiety Disord.* 31 98–107. 10.1016/j.janxdis.2015.02.003 25768399

[B13] Gold P. E. Wrenn S. M. (2012). Cycloheximide impairs and enhances memory depending on dose and footshock intensity. *Behav. Brain Res.* 233 293–297. 10.1016/j.bbr.2012.05.010 22610049 PMC3402692

[B14] Hao B. Fan B. Cao C. Liu L. Xuan S. Wang L. (2023). Genes and pathways associated with fear discrimination identified by genome-wide DNA methylation and RNA-seq analyses in nucleus accumbens in mice. *Prog. Neuropsychopharmacol. Biol. Psychiatry* 120:110643. 10.1016/j.pnpbp.2022.110643 36152737

[B15] Harney S. C. Rowan R. Anwyl R. (2006). Long-term depression of NMDA receptor-mediated synaptic transmission is dependent on activation of metabotropic glutamate receptors and is altered to long-term potentiation by low intracellular calcium buffering. *J. Neurosci.* 26 1128–1132. 10.1523/JNEUROSCI.2753-05.2006 16436598 PMC6674584

[B16] Hayashi M. (2018). Structure-function relationship of transporters in the glutamate–glutamine cycle of the central nervous system. *Int. J. Mol. Sci.* 19:1177. 10.3390/ijms19041177 29649168 PMC5979278

[B17] Herringa R. J. (2017). Trauma, PTSD, and the developing brain. *Curr. Psychiatry Rep.* 19:69. 10.1007/s11920-017-0825-3 28823091 PMC5604756

[B18] Holz A. Mülsch F. Schwarz M. Hollmann M. Döbrössy M. Coenen V. (2019). Enhanced mGlu5 signaling in excitatory neurons promotes rapid antidepressant effects via AMPA receptor activation. *Neuron* 104 338.e7–352.e7. 10.1016/j.neuron.2019.07.011 31420117

[B19] Jong Y. I. Sergin I. Purgert C. A. Malley K. L. (2014). Location-dependent signaling of the group 1 metabotropic glutamate receptor mGlu5. *Mol. Pharmacol.* 86 774–785. 10.1124/mol.114.094763 25326002 PMC4244594

[B20] Killick R. Eckley I. A. (2014). Changepoint: An R package for changepoint analysis. *J. Stat. Softw.* 58 1–19. 10.18637/jss.v058.i03

[B21] LeDoux J. E. (2014). Coming to terms with fear. *Proc. Natl. Acad. Sci. U.S.A.* 111 2871–2878. 10.1073/pnas.1400335111 24501122 PMC3939902

[B22] Lissek S. Bradford D. E. Alvarez R. P. Burton P. Espensen-Sturges T. Reynolds R. C. (2014). Neural substrates of classically conditioned fear-generalization in humans: A parametric fMRI study. *Soc. Cogn. Affect. Neurosci.* 9 1134–1142. 10.1093/scan/nst096 23748500 PMC4127021

[B23] Lissek S. Rabin S. Heller R. E. Lukenbaugh D. Geraci M. Pine D. S. (2010). Overgeneralization of conditioned fear as a pathogenic marker of panic disorder. *Am. J. Psychiatry* 167 47–55. 10.1176/appi.ajp.2009.09030410 19917595 PMC2806514

[B24] Lu Y. M. Jia Z. Janus C. Henderson J. T. Gerlai R. Wojtowicz J. M. (1997). Mice lacking metabotropic glutamate receptor 5 show impaired learning and reduced CA1 long-term potentiation (LTP) but normal CA3 LTP. *J. Neurosci.* 17 5196–5205. 10.1523/JNEUROSCI.17-13-05196.1997 9185557 PMC6573299

[B25] Lujan R. Nusser Z. Roberts J. D. Shigemoto R. Somogyi P. (1996). Perisynaptic location of metabotropic glutamate receptors mGluR1 and mGluR5 on dendrites and dendritic spines in the rat hippocampus. *Eur. J. Neurosci.* 8 1488–1500. 10.1111/j.1460-9568.1996.tb01611.x 8758956

[B26] Mayford M. Siegelbaum S. A. Kandel E. R. (2012). Synapses and memory storage. *Cold Spring Harb. Perspect. Biol.* 4:a005751. 10.1101/cshperspect.a005751 22496389 PMC3367555

[B27] Minakami R. Jinnai N. Sugiyama H. (1997). Phosphorylation and calmodulin binding of the metabotropic glutamate receptor subtype 5 (mGluR5) are antagonistic in vitro. *J. Biol. Chem.* 272 20291–20298. 10.1074/jbc.272.32.20291 9242710

[B28] Pape H. C. Pare D. (2010). Plastic synaptic networks of the amygdala for the acquisition, expression, and extinction of conditioned fear. *Physiol. Rev.* 90 419–463. 10.1152/physrev.00037.2009 20393190 PMC2856122

[B29] Parnot C. Kobilka B. (2004). Toward understanding GPCR dimers. *Mol. Biol.* 11 691–692. 10.1038/nsmb0804-691 15280880

[B30] Phillips R. G. LeDoux J. E. (1992). Differential contribution of amygdala and hippocampus to cued and contextual fear conditioning. *Behav. Neurosci.* 106 274–285. 10.1037/0735-7044.106.2.274 1590953

[B31] Poldrack R. A. Foerde K. (2008). Category learning and the memory systems debate. *Neurosci. Biobehav. Rev.* 32 197–205. 10.1016/j.neubiorev.2007.07.007 17869339

[B32] Poldrack R. A. Packard M. G. (2003). Competition among multiple memory systems: Converging evidence from animal and human brain studies. *Neuropsychologia* 41 245–251. 10.1016/S0028-3932(02)00157-4 12457750

[B33] Quadagno D. M. (1976). Intracranial cycloheximide: Effect on male mouse sexual behavior and plasma testosterone. *Pharmacol. Biochem. Behav.* 4 185–189. 10.1016/0091-3057(76)90013-7 1265105

[B34] Rahman M. M. Kedia S. Fernandes G. Chattarji S. (2017). Activation of the same mGluR5 receptors in the amygdala causes divergent effects on specific versus indiscriminate fear. *Elife* 6:e25665. 10.7554/eLife.25665 28555566 PMC5468087

[B35] Schulz B. Fendt M. Gasparini F. Lingenhöhl K. Kuhn R. Koch M. (2001). The metabotropic glutamate receptor antagonist 2-methyl-6-(phenylethynyl)-pyridine (MPEP) blocks fear conditioning in rats. *Neuropharmacology* 41 1–7. 10.1016/S0028-3908(01)00036-3 11445180

[B36] Shallcross J. Wu L. Wilkinson C. S. Knackstedt L. A. Schwendt M. (2021). Increased mGlu5 mRNA expression in BLA glutamate neurons facilitates resilience to the long-term effects of a single predator scent stress exposure. *Brain Struct. Funct.* 226 2279–2293. 10.1007/s00429-021-02326-4 34175993 PMC10416208

[B37] Sun Y. Gooch H. Sah P. (2020). Fear conditioning and the basolateral amygdala. *F1000Res.* 9:53. 10.12688/f1000research.21201.1 32047613 PMC6993823

[B38] Tronson N. C. Guzman Y. F. Guedea A. L. Huh K. H. Gao C. Schwarz M. K. (2010). Metabotropic glutamate receptor 5/Homer interactions underlie stress effects on fear. *Biol. Psychiatry* 68 1007–1015. 10.1016/j.biopsych.2010.09.004 21075228 PMC2987592

[B39] Wang H. MacDonald M. L. Borgmann-Winter K. E. Banerjee A. Sleiman P. Tom A. (2020). MGluR5 hypofunction is integral to glutamatergic dysregulation in schizophrenia. *Mol. Psychiatry* 25 750–760. 10.1038/s41380-018-0234-y 30214040 PMC7500805

[B40] Wiltgen B. J. Silva A. J. (2007). Memory for context becomes less specific with time. *Learn. Mem.* 14 313–317. 10.1101/lm.430907 17522020

[B41] Xu J. Zhu Y. Contractor A. Heinemann S. F. (2009). MGluR5 has a critical role in inhibitory learning. *J. Neurosci.* 29 3676–3684. 10.1523/JNEUROSCI.5716-08.2009 19321764 PMC2746052

[B42] Xu W. Südhof T. C. (2013). A neural circuit for memory specificity and generalization. *Science* 339 1290–1295. 10.1126/science.1229534 23493706 PMC3651700

[B43] Zaki S. R. Nosofsky R. M. Jessup N. M. Unverzagt F. W. (2003). Categorization and recognition performance of a memory-impaired group: Evidence for single-system models. *J. Int. Neuropsychol. Soc.* 9 394–406. 10.1017/S1355617703930050 12666764

[B44] Zhou H. Xiong G. Jing L. Song N. Pu D. Tang X. (2017). The interhemispheric CA1 circuit governs rapid generalisation but not fear memory. *Nat. Commun.* 8:2190. 10.1038/s41467-017-02315-4 29259187 PMC5736595

