# Supplementary material for: mGluR5 in amygdala modulates fear memory generalization
Source: Front Behav Neurosci. 2023 Feb 20;17:1072642. doi: 10.3389/fnbeh.2023.1072642 (PMC9986332; doi:10.3389/fnbeh.2023.1072642)
Supplement: Supplementary file 1 [file Data_Sheet_1.docx]

Supplementary Material

# Supplementary Figures

**Supplementary Figure S1.** (A) Representative activity tracking of CTR and hM4Di mice in OF. (B) Total distance traveled, total active time, time in centre region, and distance in center region percentage during OFT(unpaired two-tailed Student t test, Two-tailed, total distance, t=0.1834, df=14, *p*= 0.8574; centre distance/total distance, t=0.2320, df=14, p= 0.8199; total active time, t=0.4190, df=14, *p*= 0.6816; centre time, t=1.008, df=14, *p*= 3306).

**Supplementary Figure S2.** (A) p-GluN2 and GS proteins levels in the synaptic fraction at different time point after fear conditioning. (B) p-GluN2 level is decreased at 8h and 24h after fear conditioning (p-GluN2, one-way ANOVA, 0h: F2, 9= 1.065, *p*=0.3846; 8h: F2,9= 5.694, *p*=0.0252, multiple comparison, Fisher’s LSD test, *p*[Control vs WS]= 0.0198, *p*[Control vs SS]= 0.0147, *p*[WS vs SS]= 0.9822; 24h: F2,9= 8.939, *p*=0.0073, multiple comparison, Fisher’s LSD test, *p*[Control vs WS]= 0.0051, *p*[Control vs SS]= 0.053, *p*[WS vs SS]= 0.9797. GS, one-way ANOVA, 0h: F2, 9= 1.779, *p*=0.2233; 8h: F2, 9= 1.073, *p*=0.3820; 24h: F2, 9 = 0.1203, *p*=0.8880).

**Supplementary Figure S3.** (A) HE staining image shows that cannulas are implanted above amygdala. (B) Representative activity tracking of saline and MPEP mice in OFT. (C) Total distance traveled, total active time, time in center region, and distance in center region percentage during OFT (unpaired two-tailed Student t test, Two-tailed, total distance, t=1.479, df=6, *p*= 0.1896; centre distance/total distance, t=0.8356, df=6, *p*= 0.4354; total active time, t=1.040, df=6, *p*= 0.3383; centre time, t=1.622, df=6, *p*= 0.1560).

# Supplementary Table

| Gene | Forward primer （5'->3'） | Reverse primer （5'->3'） |
| --- | --- | --- |
| Ncdn | GGTTCACCTTTCCCAACCGA | GCTGGCTAGTTCAGGGTCG |
| Tamalin | TGGAGGACTATCACCCTGCC | TTCCGTTGCTGTTCAGGACTC |
| Frmpd4 | ACCGAGATGGACGTGACTACT | TGGTGTTACTGACCGAACGAC |
| Homer1 | CCCGATGTGACACAGAACTCA | AGCTCTCCAGCAAAGAAAGTTG |
| Pik3r1 | ACACCACGGTTTGGACTATGG | GGCTACAGTAGTGGGCTTGG |
| Actin | CTCTCCCTCACGCCATC | ACGCACGATTTCCCTCTC |
| GRM5 | ACCAACCAACTGTGGACAAAG | CAAGAGTGTGGGATCTGAATTGA |
