# Supplementary figures and images for: mGluR5 in amygdala modulates fear memory generalization
Source: Front Behav Neurosci. 2023 Feb 20;17:1072642. doi: 10.3389/fnbeh.2023.1072642 (PMC9986332; doi:10.3389/fnbeh.2023.1072642)

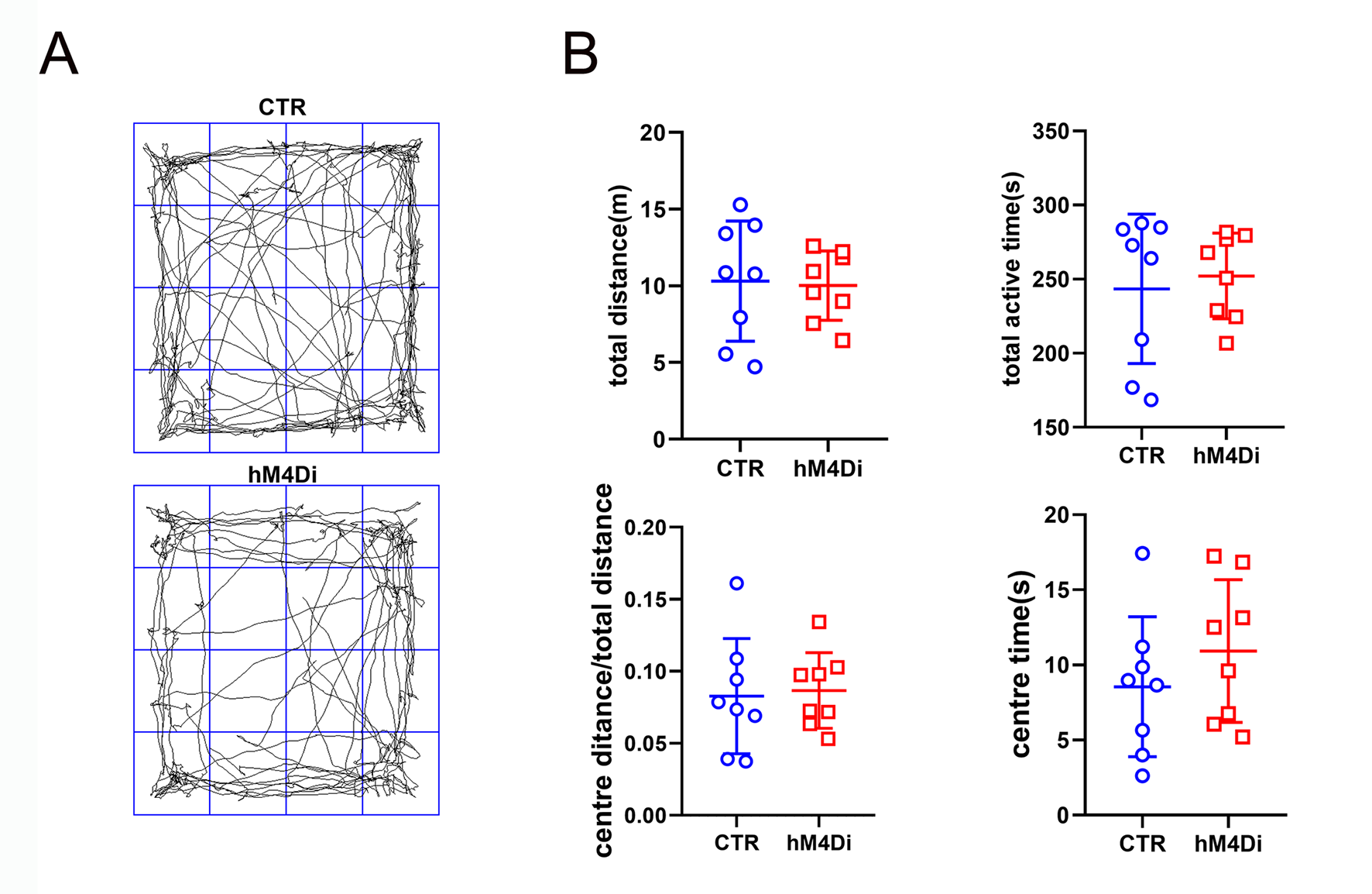

Supplement: Supplementary file 2 [file Image_1.TIF]

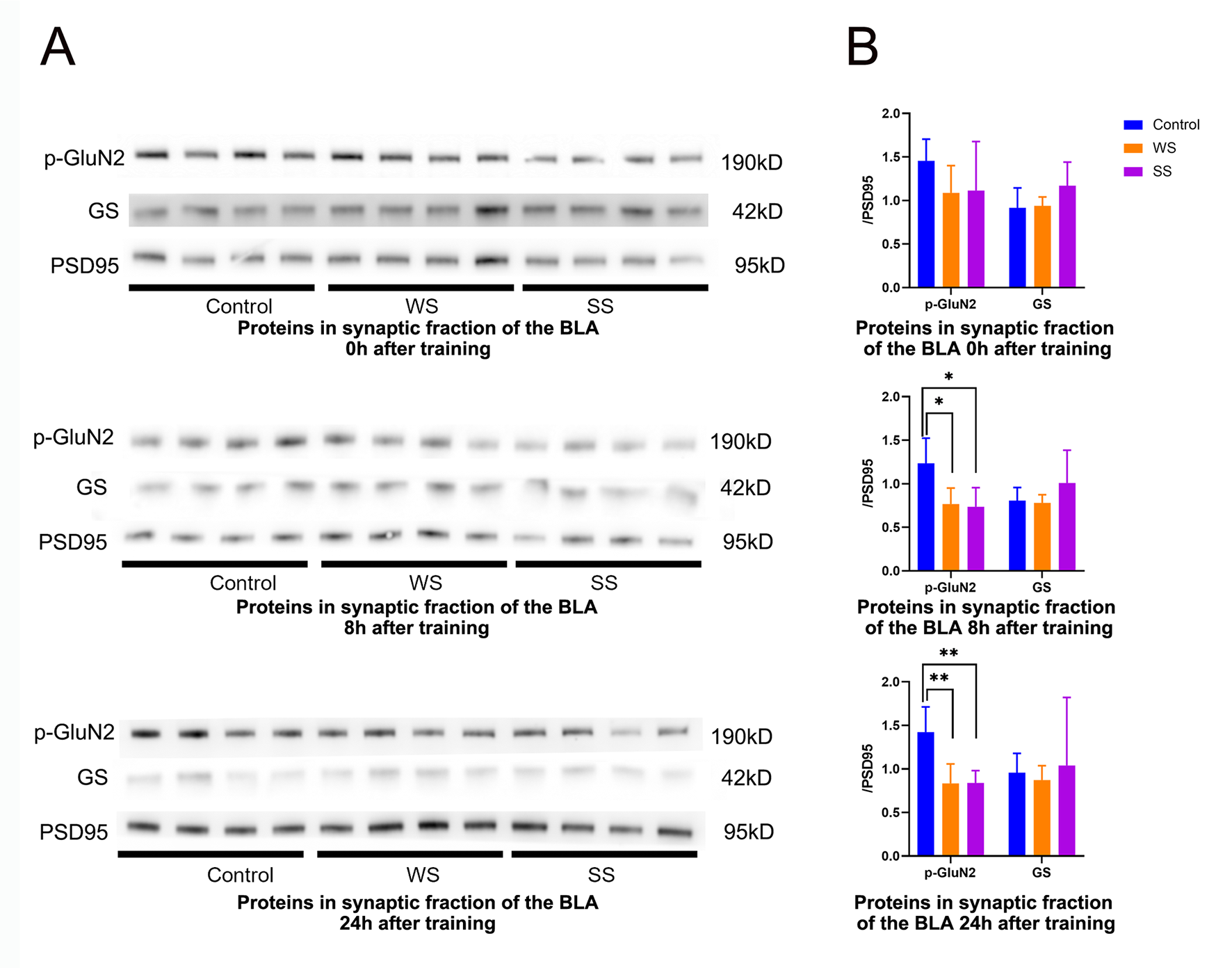

Supplement: Supplementary file 3 [file Image_2.TIF]

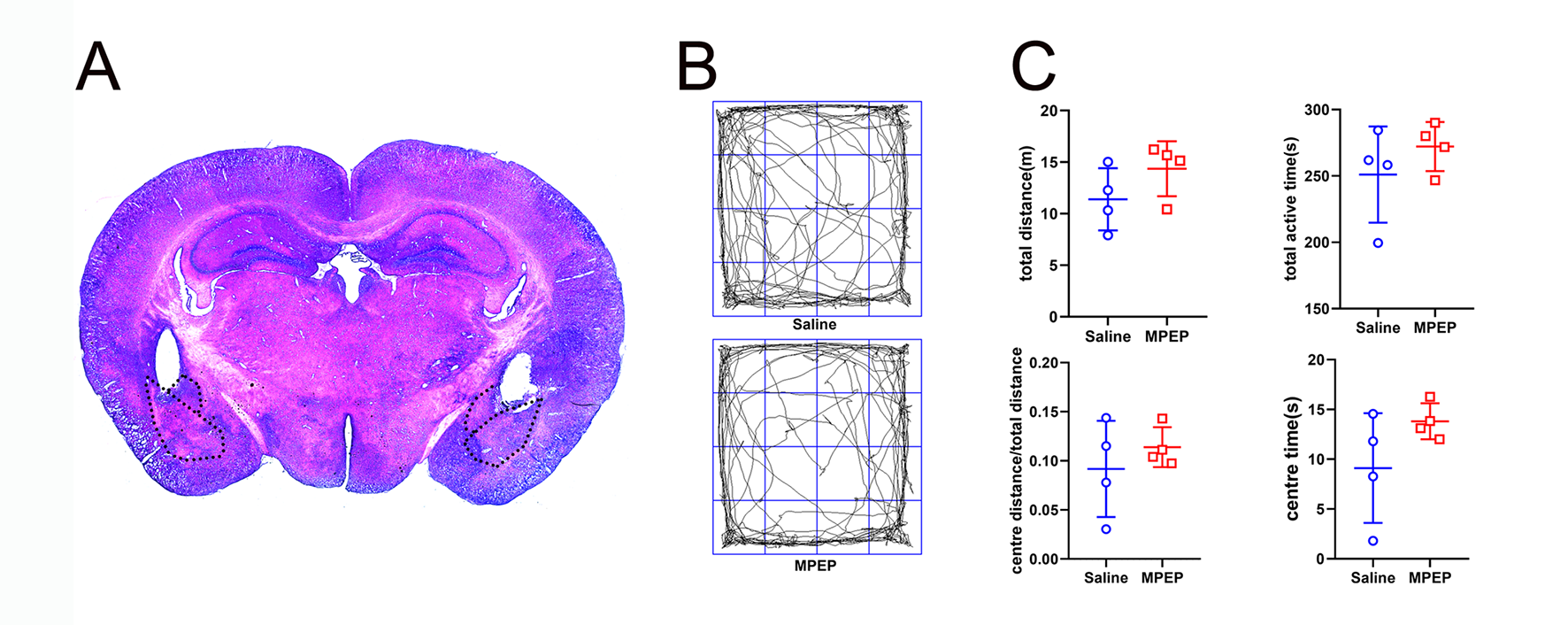

Supplement: Supplementary file 4 [file Image_3.TIF]
